# Effect of HCG-Triggered Ovulation on Pregnancy Outcomes in Intrauterine Insemination: An Analysis of 5,610 First IUI Natural Cycles With Donor Sperm in China

**DOI:** 10.3389/fendo.2020.00423

**Published:** 2020-07-07

**Authors:** Ji-Peng Wan, Zhen-Jing Wang, Yan Sheng, Wei Chen, Qing-Qing Guo, Jin Xu, Hua-Rui Fan, Mei Sun

**Affiliations:** ^1^Department of Obstetrics and Gynecology, Cheeloo College of Medicine, Shandong Provincial Hospital, Shandong University, Jinan, China; ^2^Department of Obstetrics and Gynecology, Shandong Provincial Hospital Affiliated to Shandong First Medical University, Jinan, China; ^3^Center for Reproductive Medicine, Cheeloo College of Medicine, Shandong University, Jinan, China; ^4^Key Laboratory of Reproductive Endocrinology of Ministry of Education, National Research Center for Assisted Reproductive Technology and Reproductive Genetics, Shandong University, Jinan, China

**Keywords:** IUI, hCG, LH, pregnancy, ovulation

## Abstract

**Objective:** To evaluate the effect of human chorionic gonadotropin (hCG) trigger ovulation on pregnancy outcomes in natural IUI cycles with donor sperm.

**Methods:** This retrospective cohort study included 5,610 first-natural IUI cycles with donor sperm in infertile couples during the period from January 2012 to December 2017. To control for other confounding factors, our analysis was restricted to normo-ovulatory women without tubal infertility. The main outcome measure was live birth rate; the secondary outcomes included rates of clinical pregnancy and miscarriage.

**Results:** In the crude analysis, both the clinical pregnancy (27.40 vs. 22.73%; *P* = 0.001) and live birth rates (24.52 vs. 20.13%; *P* = 0.007) were significantly higher for the hCG group than for the spontaneous LH group. After adjustment for a number of confounding factors, the reproductive outcomes were still significantly worse for the spontaneous ovulatory group.

**Conclusions:** Among women undergoing natural cycle IUI with donor sperm, hCG triggered ovulation for timing insemination offers beneficial impacts on both clinical pregnancy rates and live birth rates.

## Introduction

Intrauterine insemination (IUI), a minimally invasive and low-cost procedure, is a first-line treatment for a broad range of indications in reproductive medicine (1, 2). IUI involves timely scheduled insemination of sperm into the uterus, either in natural cycles, or following ovarian stimulation. Compared to stimulated IUI, natural cycle IUI is preferable for patients because of advantages including lower medication cost and reduced rates of multiple gestation (3).

It is known that the timing of insemination is one of the most impactful factors influencing the success rates of IUI (4, 5). There are various methods for timing IUI, and administration of human chorionic gonadotropin (hCG) is widely accepted in clinical practice. Administration of hCG requires less endocrine monitoring, but its effect on endometrial receptivity has drawn extensive attention recently (6–8).

Multiple clinical studies have assessed the effect of hCG on pregnancy outcomes, but have reached inconsistent conclusions (9–12). Such conflicting results result from clinical indication, female age, baseline characteristics of study participants, sperm quality, timing, or type of ovarian stimulation.

The current study examined a large cohort of women undergoing natural cycle IUI and aimed to assess the effect of hCG-induced ovulation on pregnancy outcome of natural cycle IUI. Attempting to control for sperm-related factors, this retrospective study only examined patients using donor sperm.

## Materials and Methods

### Study Design

This retrospective study included women who underwent IUI with donor sperm in a natural menstrual cycle. The trial was conducted between January 2012 and December 2017 at the Center for Reproductive Medicine of the Shandong Provincial Hospital Affiliated to Shandong University. The Institutional Review Board approved the research project.

Prior to the IUI cycles, all women underwent basic fertility investigation that included evaluations of the following: cycle day 3 FSH, LH, estradiol, anti-Müllerian hormone (AMH), prolactin, and thyroid stimulating hormone (TSH) levels, hysterosonography (at least 1 month prior to IUI), as well as transvaginal ultrasound for evaluation of pelvic anatomy and follicular monitoring.

Study inclusion criteria were: age ≤35 years, regular menstrual cycles (28–35 days), no personal history of infertility or medical conditions likely to cause infertility, a normal baseline hormonal profile, and patency of at least one fallopian tube. Exclusion criteria were the presence of irregular menstrual cycles, tubal factor infertility, confirmed endometriosis, a diagnosis of polycystic ovarian syndrome (according to the Rotterdam criteria; Rotterdam ESHRE/ASRM-Sponsored PCOS Consensus Workshop Group, 2004), abnormal TSH or prolactin levels, or any known metabolic or endocrinological disease. Women with incomplete medical records were excluded from the study, as were women who took any medication.

The choice to use hCG trigger or urinary LH monitoring plus transvaginal ultrasonography was made according to the patient's preference and availability for monitoring. A home urinary ovulation prediction kit was used to monitor urinary LH levels once a day, in the evening around 19:00 h, starting on cycle day 10. And serial ultrasonography for follicular monitoring was also started on cycle day 10 for all cycles. When ovulation was diagnosed, insemination was performed immediately.

In the spontaneous ovulation group (based on urinary LH monitoring and transvaginal ultrasonography), insemination was carried out within 24 h after detection of the first urinary LH positive test or upon detection of ultrasound features suggestive of ovulation. In the induced ovulation group (administration of hCG trigger), when a leading follicle reached a diameter of 16 mm or urinary LH surge appeared, ovulation was triggered using 10,000 IU of recombinant hCG (choriogonadotropin alfa, Ovidrel® EMD Serono, Inc.). In our practice, the standard of care is to perform two inseminations in a given cycle, approximately 24 and 36 h after HCG injection (13–15); however, there are clinical factors affecting the clinical decision to use single or double IUI including patient demand, financial barriers, or ovulation time.

Frozen donor sperm were obtained from the Shandong Province Human Sperm Bank of China, and were thawed on site on the day of insemination. All sperm samples were prepared with a density gradient centrifugation method using Puresperm TM (Nidacon, International AB) (16). The quality of semen was evaluated before and after processing (semen TMS).

The insemination procedure was the same for all women, bed rest was maintained for 15 min after the insemination (17). A urine pregnancy test was administered 14 days after insemination, and if positive, a transvaginal ultrasound would be performed 14 days later to assess the pregnancy outcome (including the number of gestational sacs, viability, and location).

### Data Analyses

Clinical pregnancies were confirmed by the presence of a fetal heartbeat on ultrasound scanning 7 weeks after insemination. Miscarriage was defined as loss of a clinical pregnancy before the 28th week of gestation. Live birth was defined as delivery of a live neonate after 28 weeks' gestation. Our primary outcome measure was live birth rate. Our secondary outcomes included clinical pregnancy rate and miscarriage rate. In light of the significant differences detected related to intrauterine insemination number between the hCG group and the urinary LH group, a subgroup analysis was performed to assess the impacts of one vs. two inseminations in a given cycle for both the hCG and urinary LH groups.

Statistical analysis was performed using SPSS Statistics 20.0 (IBM). Mean values and standard deviations were calculated for each continuous variable, whereas percentages were determined for the categorical variables. Continuous variables were compared with Student's *t*-tests. Categorical variables were compared with chi-squared and Fisher's exact tests. A single, multivariate logistic regression analysis (incorporating ovulation timing method as a categorical explanatory variable) was performed to assess the independent effect of hCG administration on reproductive outcomes after adjustment for possible confounding factors, including maternal age, BMI, number of intrauterine insemination events, and basal hormone levels (FSH, LH, estradiol, AMH). A *P* < 0.05 was considered statistically significant.

## Results

A total of 5,610 patients who were treated with IUI from January 2012 until December 2017 were included in the study. Despite the fact that some women received several IUI treatments per fertility cycle, only data for the first treatment (either single or double insemination) of each woman was included in the analysis.

The baseline characteristics and hormonal profiles of the patients in the hCG and spontaneous ovulation group are presented in Table 1. No significant differences were observed regarding BMI, basal AMH, or estradiol concentration between patients of the two groups, while slight differences were detected in age and basal FSH and basal LH concentrations.

**Table 1:** Demographic characteristics of the patient population.

**Characteristics**	**HCG administration**	**Spontaneous ovulation**	***P***
Patients, n	4,343	1,267	
Age (years)	27.25 ± 3.40	27.51 ± 3.29	0.014
BMI (kg/m^2^)	22.11 ± 3.15	22.14 ± 3.19	0.864
AMH(ng/ml)	4.20 ± 2.90	4.28 ± 2.93	0.54
FSH (IU/L)	6.64 ± 1.77	6.83 ± 1.82	0.001
LH (mIU/ml)	4.88 ± 2.39	5.07 ± 2.16	0.012
Estradiol (pg/mL)	34.75 ± 13.17	34.25 ± 12.69	0.617
Endometrium thickness on the day of insemination (cm)	1.01 ± 0.11	1.02 ± 0.14	0.06
IUI number	1.89 ± 0.31	1.29 ± 0.45	<0.001

*Data are presented as mean ± SD for continuous variables*.

*BMI, body mass index; AMH, anti-Müllerian hormone; IUI, intrauterine insemination*.

Pregnancy outcomes are shown in Table 2. In crude analysis, compared to the spontaneous ovulation group, the hCG group had significantly higher rates of clinical pregnancy [27.40 vs. 22.73%, OR (95% CI): 1.28 (1.11–1.49); *P* = 0.001] and live birth rates [24.52 vs. 20.13%, OR (95% CI); 1.29 (1.11–1.50); *P* = 0.001]. Additionally, no difference in the rate of miscarriage was observed between the two groups [10.50 vs. 11.46%, OR (95% CI): 0.91(0.60–1.36); *P* = 0.671]. Note that the significantly increased clinical pregnancy and live birth rates of the hCG group were still detected after adjustment for a number of confounding factors (Table 2).

**Table 2:** Reproductive outcomes of the patient population.

**Characteristics**	**HCG administration**	**Spontaneous ovulation**	**OR (95% CI)**	**AOR (95% CI)**	***P***	***P* adj**
Clinical pregnancy	1,190/4,343 (27.40%)	288/1,267 (22.73%)	1.28 (1.11 1.49)	1.26 (1.05 1.51)	0.001	0.013
Miscarriage	125/1,190 (10.50%)	33/288 (11.46%)	0.91 (0.60 1.36)	0.75 (0.46 1.23)	0.671	0.255
Live birth	1,065/4,343 (24.52%)	255/1,267 (20.13%)	1.29 (1.11 1.50)	1.30 (1.07 1.57)	0.001	0.007

*Data are presented as n/n (%) for dichotomous variables OR Odd ratio, AOR adjusted odd ratio*.

*Clinical pregnancies were confirmed by the presence of a fetal heartbeat on ultrasound scanning 7 weeks after insemination. Miscarriage was defined as loss of a clinical pregnancy before the 28th week of gestation. Live birth was defined as delivery of a live neonate after 28 weeks' gestation*.

*Analyses were adjusted for maternal age, BMI, number of intrauterine insemination events, and basal hormone levels (FSH, LH, estradiol, AMH)*.

To assess the effect of intrauterine insemination number on pregnancy outcomes, a subgroup analysis (single IUI, double IUI) was performed (Tables 3, 4). Among women who received double IUI, there was no difference between the hCG group and spontaneous ovulation group in the rates of clinical pregnancy [27.30 vs. 25.00%, OR (95% CI): 1.12 (0.88–1.44); *P* = 0.343] or live birth [24.46 vs. 20.66%, OR (95% CI): 1.24 (0.96–1.62); *P* = 0.102]. However, among single IUI patients, the hCG group women achieved significantly higher rates of clinical pregnancy [28.23 vs. 21.80%, OR (95% CI): 1.41 (1.09–1.81); *P* = 0.009] and live birth [25.00 vs. 19.91%, OR (95% CI): 1.34 (1.03–1.74); *P* = 0.032].

**Table 3:** Reproductive outcomes of the single IUI group.

**Characteristics**	**HCG administration**	**Spontaneous ovulation**	**OR (95% CI)**	**AOR (95% CI)**	***P***	***P* adj**
Clinical pregnancy	131/464 (28.23%)	196/899 (21.80%)	1.41 (1.09 1.81)	1.39 (1.08 1.81)	0.009	0.011
Miscarriage	15/132 (11.36%)	17/196 (8.67%)	1.35 (0.65 2.81)	1.27 (0.60 2.67)	0.421	0.530
Live birth	116/464 (25.00%)	179/899 (19.91%)	1.34 (1.03 1.74)	1.34 (1.02 1.74)	0.032	0.034

*Data are presented as n/n (%) for dichotomous variables OR Odd ratio, AOR adjusted odd ratio*.

*Clinical pregnancies were confirmed by the presence of a fetal heartbeat on ultrasound scanning 7 weeks after insemination. Miscarriage was defined as loss of a clinical pregnancy before the 28th week of gestation. Live birth was defined as delivery of a live neonate after 28 weeks' gestation*.

*Analyses were adjusted for maternal age, BMI, number of intrauterine insemination events, and basal hormone levels (FSH, LH, estradiol, AMH)*.

**Table 4:** Reproductive outcomes of the double IUI group.

**Characteristics**	**HCG administration**	**Spontaneous ovulation**	**OR (95% CI)**	**AOR (95% CI)**	***P***	***P* adj**
Clinical pregnancy	1,059/3,879 (27.30%)	92/368 (25.00%)	1.12 (0.88 1.44)	1.12 (0.88 1.44)	0.343	0.356
Miscarriage	110/1,059 (10.39%)	16/92 (17.39%)	0.55 (0.31 0.99)	0.52 (0.29 0.94)	0.039	0.029
Live birth	949/3,879 (24.46%)	76/368 (20.66%)	1.24 (0.96 1.62)	1.25 (0.96 1.62)	0.102	0.103

*Data are presented as n/n (%) for dichotomous variables OR Odd ratio, AOR adjusted odd ratio*.

*Clinical pregnancies were confirmed by the presence of a fetal heartbeat on ultrasound scanning 7 weeks after insemination. Miscarriage was defined as loss of a clinical pregnancy before the 28th week of gestation. Live birth was defined as delivery of a live neonate after 28 weeks' gestation*.

*Analyses were adjusted for maternal age, BMI, number of intrauterine insemination events, and basal hormone levels (FSH, LH, estradiol, AMH)*.

## Discussion

In this study, we found that administration of hCG for triggering ovulation was associated with increased pregnancy rates in IUI with donor sperm in a natural menstrual cycle. When we divided the patients into subgroups according to IUI number, the increased pregnancy rate in the hCG group was only observed in the single IUI subgroup (18).

Previous research has established that the success of IUI depends on various factors, including maternal age, sperm quality, type of subfertility, ovarian stimulation, and the timing of insemination (19, 20). Given that spermatozoa and oocytes have only limited survival times, the appropriate timing of IUI relative to ovulation may be one of the most important factors influencing IUI success (6). Ovulation typically occurs 25–56 h following the onset of a spontaneous LH surge, whereas ovulation usually occurs 36–48 h after hCG administration in natural cycles.

Administration of hCG makes clinical prediction of ovulation more accurate; it permits planning for optimized time intervals between ovulation and insemination possible (4). This increased accuracy can likely help explain the significantly increased pregnancy rates achieved by the hCG group women in the present study. When insemination was performed twice, the window of sperm exposure to the oocyte was significantly increased; this maybe help explain the similar clinical pregnancy rates observed for the two groups (21).

Several studies of ovarian hyperstimulation IUI cycles have demonstrated beneficial effects of hCG administration on IUI pregnancies (22–24). One study by Taerk et al. reported that hCG administration significantly increased clinical pregnancy rates compared with monitoring of spontaneous serum LH surge in subfertile patients undergoing controlled ovarian hyperstimulation IUI cycles (22). Two additional retrospective studies of stimulated IUI have also reported that higher pregnancy rates resulted when hCG was given to trigger ovulation (23, 24).

Few studies have explored association(s) between hCG administration and pregnancy outcomes in natural cycle IUI. Moreover, the few studies addressing this topic have yielded inconsistent results. A Cochrane meta-analysis by Cantineau et al. reported no difference in pregnancy rates or live birth rates between natural cycle IUI patients timed according to spontaneous LH surge monitoring or hCG triggering (25). However, note that these reports included a total of only 264 women, and only one study reported live birth rate data.

To date, only one randomized clinical trial has investigated the pregnancy outcome of hCG administration for triggering ovulation in natural cycle IUI (9). A total of 300 patients were included in that study, of which 197 women used donor sperm. The trial concluded that administration of hCG resulted in decreased ongoing pregnancy rates. Given that the goal of IUI treatment is to achieve a healthy live birth, it is unfortunate that live birth rate data for the two groups was not reported from the trial.

A retrospective cohort study by El Hachem et al. found no difference in clinical pregnancy or live birth rates between urinary LH monitoring vs. hCG-triggered ovulation in natural unstimulated therapeutic donor sperm insemination cycles (11). The inclusion criteria of that study were strict: only normo-ovulatory women were included. Notably, whereas the average age of the patients in that study was 32, the mean age of patients in our study was 27 years. We suspect that this 5 years age gap may be a major factor underlying the inconsistent conclusions of the two studies.

Limitations of our study include the use of urinary LH monitoring, which has been associated with increased probability for false-negative results; such results would cause inaccurate timing and thereby likely contribute to decreased pregnancy rates (3). However, considering its advantages over alternatives (like serum LH testing) such as ease-of-use, non-invasiveness, and low cost, urinary LH monitoring is widely used in the clinic (26, 27). It also bears mention that our clinical pregnancy rate in the spontaneous LH group was 22.73%, a rate comparable to previously published natural cycle IUI success rates. Second, our study is based on a retrospective design. The inclusion criteria were strict, with only normo-ovulatory women aged ≤35 years who underwent IUI with high-quality donor sperm included, which enabled analysis of a well-defined cohort of women with no obvious confounding factors. The current study also had several strengths, the foremost of which was its large cohort size. To the best of our knowledge, ours is the largest-to-date study comparing the two major IUI timing methods for natural cycle IUI. Moreover, our study monitored the live birth rate of natural cycle IUI, data which has been reported rarely in previous studies.

In conclusion, the administration of hCG for triggering of ovulation is associated with significantly higher pregnancy rates compared with spontaneous ovulation in patients undergoing natural IUI. Thus, using hCG for timing ovulation optimizes the chances of success for natural cycle IUI with donor sperm.

## Data Availability Statement

All datasets generated for this study are included in the article/supplementary material.

## Ethics Statement

The studies involving human participants were reviewed and approved by The Institutional Review Board, Shandong Provincial Hospital Affiliated to Shandong University, Jinan, China, and gave the ethical approval for the study (Number 6166). The patients/participants provided their written informed consent to participate in this study.

## Author Contributions

J-PW and Z-JW: conceptualization. H-RF: data curation. MS: investigation. WC and Q-QG: methodology. YS and JX: project administration. J-PW: writing—original draft. Z-JW and MS: writing—review & editing. All authors participated in the ultimate interpretation of the study data and in revisions to the article.

## Conflict of Interest

The authors declare that the research was conducted in the absence of any commercial or financial relationships that could be construed as a potential conflict of interest.
